# Editorial: Innovative and cutting-edge approaches to the identification and management of autism spectrum disorders

**DOI:** 10.3389/fpsyt.2026.1794305

**Published:** 2026-02-19

**Authors:** Sabri Hergüner

**Affiliations:** Child and Adolescent Psychiatry Section, Ankara Autism Center, Ankara, Türkiye

**Keywords:** autism, autism spectrum disorder, identification, innovation, management

Autism Spectrum Disorder (ASD) remains one of the most complex and clinically significant neurodevelopmental conditions of our time. Despite major advances in research and clinical practice, challenges persist throughout the entire pathway of care: from timely identification and differential diagnosis to individualized intervention and long-term outcomes. This Research Topic, *“Innovative and Cutting-edge Approaches to the Identification and Management of Autism Spectrum Disorders”*, brings together a set of studies that reflect a shared vision: bridging technological innovation with real-world clinical relevance, while advancing our ability to understand autism not only behaviorally, but also physiologically and functionally across development.

A central theme of this Research Topic is the pursuit of objective biomarkers and technology-assisted assessments, which have the potential to complement established diagnostic standards and reduce reliance on subjective interpretations alone. Several contributions in this Research Topic aim to address this gap by introducing automated, data-driven approaches capable of quantifying key behavioral or neurophysiological features of ASD with promising accuracy.

The studies in this Research Topic collectively map a compelling landscape of innovation in ASD identification and management. From robotics and EEG biomarkers to explainable machine learning to immersive interventions, music therapy, neuromodulation, cognitive profiling, and integrated AI platforms, the contributions reflect a shared commitment to advancing both science and service delivery. The field of autism research stands at a moment where technological progress is rapid, but translation into everyday clinical reality remains uneven. The works presented here help narrow this gap. It also reminds us that “cutting-edge” innovation is most meaningful when it is grounded in clinical need, guided by scientific rigor, and measured against outcomes that matter to autistic individuals and their families.

